# Ca-Dependent Folding of Human Calumenin

**DOI:** 10.1371/journal.pone.0151547

**Published:** 2016-03-18

**Authors:** Marco Mazzorana, Rohanah Hussain, Thomas Sorensen

**Affiliations:** Diamond Light Source, Ltd, Life Sciences Division, Harwell Science and Innovation Campus, Didcot, United Kingdom; University of Newcastle, AUSTRALIA

## Abstract

Human calumenin (hCALU) is a six EF-hand protein belonging to the CREC family. As other members of the family, it is localized in the secretory pathway and regulates the activity of SERCA2a and of the ryanodine receptor in the endoplasmic reticulum (ER). We have studied the effects of Ca^2+^ binding to the protein and found it to attain a more compact structure upon ion binding. Circular Dichroism (CD) measurements suggest a major rearrangement of the protein secondary structure, which reversibly switches from disordered at low Ca^2+^ concentrations to predominantly alpha-helical when Ca^2+^ is added. SAXS experiments confirm the transition from an unfolded to a compact structure, which matches the structural prediction of a trilobal fold. Overall our experiments suggest that calumenin is a Ca^2+^ sensor, which folds into a compact structure, capable of interacting with its molecular partners, when Ca^2+^ concentration within the ER reaches the millimolar range.

## Introduction

**Fig 1:**
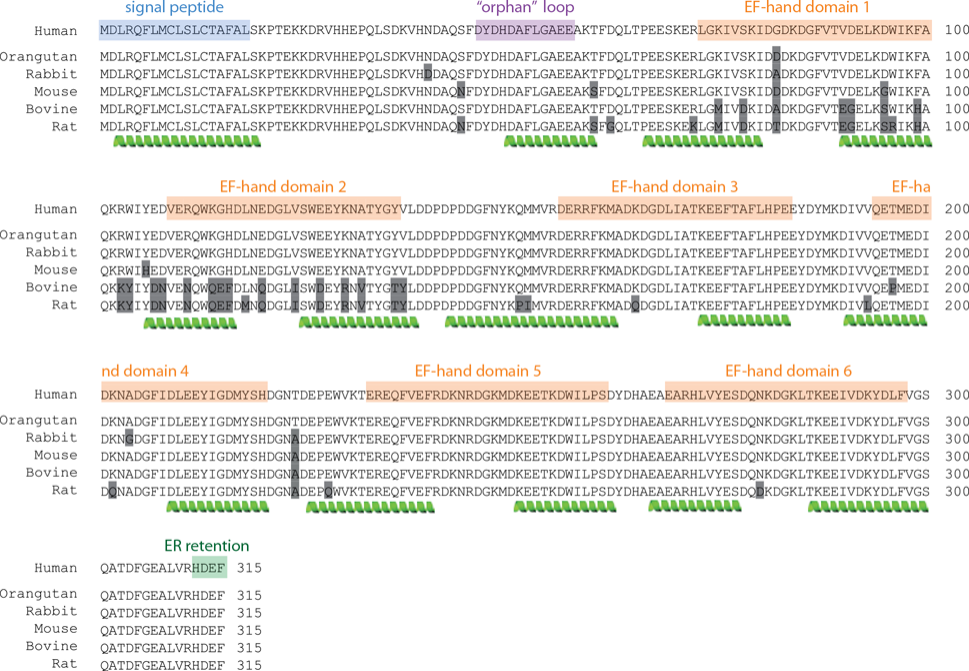
Annotated sequence of calumenin. Primary structure of calumenin with predicted secondary structure elements (alpha-helices indicated in green below amino acid sequence). Six canonical EF-hand domains are identified (orange boxes) located between an N-terminal ER signal peptide (aa. 1–19) and a C-terminal ER retention sequence (HDEF). An “orphan loop” (aa. 46–58), lacking the flanking alpha-helices found in EF-domains is identified as an additional Ca^2+^-binding motif.

Calumenin is a 37 kDa protein belonging to the CREC protein family (acronym derived from the four main family members: Cab45, reticulocalbin1, ERC-55 and calumenin [1]). These proteins have multiple EF-hand motifs [2] and have been characterised as low-affinity Ca^2+^ binders distributed along the secretory pathway [3] (Fig 1 and S1 Fig).

Calumenin is expressed ubiquitously in human cells, but the highest mRNA levels have been found in smooth muscle and cardiomyocytes [4]. The protein resides inside the endoplasmic reticulum (ER), directed there by an N-terminal signal peptide as well as by a unique C-terminal retention sequence [5].

Analysis of the primary structure highlights the presence of six EF-hand motifs, with the possible existence of a further seventh degenerate Ca^2+^ binding loop [6], which we refer to as a “orphan” loop. This retains the dominants for coordinating Ca^2+^, but lacks the flanking alpha-helical structures to form an EF-hand domain.

Results from previous studies suggest that calumenin has a low Ca^2+^ affinity and binds up to 7 Ca^2+^ ion with K_d_ in the low mM range [6], consistent with similar findings for other Ca^2+^-binding proteins [7] located within the ER. This is in agreement with the much higher concentrations of Ca^2+^ needed to activate ER-located proteins compared to their cytosolic counterparts [3]. In general, Ca^2+^-binding proteins coordinate the ion through aspartate and glutamate residues. Not all however bind Ca^2+^ to exposed loops such as the EF-hand proteins. Calsequestrin, a low-affinity Ca^2+^-binding protein, coordinates up to 50 Ca^2+^ ions through residues on its highly negatively charged surface to buffer Ca^2+^ in the ER lumen. This high-capacity Ca^2+^-binding protein also provides indirect control on the homeostasis of Ca^2+^ by polymerising in the presence of mM concentrations of the ion to regulate the activity of the ryanodine receptor [7].The lower Ca^2+^-binding capacity of calumenin suggests that it does not contribute significantly to Ca^2+^ buffering, but primarily acts as a Ca^2+^ sensor regulating several Ca^2+^-dependent processes occurring inside the ER (*e*.*g*. protein folding and maturation) [8]. Calumenin possibly also interacts directly with proteins involved in Ca^2+^ homeostasis, in particular the Sarco(Endoplasmic)Reticulum Ca^2+^-ATPase (SERCA2a) [9] and the ryanodine receptor [10], thereby regulating their function. Calumenin can therefore be considered a key player in ER Ca^2+^ homeostasis and given its high expression level in myocytes, it is likely to play a fundamental role in the control of muscle contraction/relaxation both in smooth muscle and in heart (reviewed in [11]).

So far very little is known about the structure-function relationship of calumenin in response to variation of Ca^2+^ concentration. In this study we have analysed the Ca^2+^ -dependent structural behaviour of calumenin and propose a folding model that provides a structural explanation of Ca^2+^ sensing–a feature likely to be shared across the CREC family.

## Materials and Methods

### Protein expression and purification

The region encompassing the 6 EF-hand repeats of human calumenin isoform 1 (Uniprot O43852, amino-acids 68–315) was cloned in the vector pOPINS as previously described [12]. Rosetta (DE3) *E*. *coli* cells (Novagen) were used for expression starting from a saturated overnight culture in LB-Miller broth supplemented with 0.1% glucose. The protein was expressed from 1 litre of LB-Miller broth supplemented with autoinduction additives [13], inoculated with 10 ml of the overnight culture and kept 5 hours at 37°C before lowering the temperature to 18°C for 60 hours. Cells were harvested by centrifugation and stored at -80°C.

Approximately 40 g of frozen bacterial cell pellet were suspended in buffer A (25 mM Na-HEPES pH 7.5, 500 mM NaCl, 25 mM Imidazole, 2.5 mM CaCl_2_, 0.1% α-monothyoglycerol) supplemented with mini EDTA-free protease inhibitors (Roche). Cells were lysed by 2 passes on a Constant Systems TS Cell Disruptor at 4°C and a pressure of 30 kPsi. Lysate was clarified by centrifugation (45 minutes at approx. 39000 xg) and applied on a 5 ml HisTrapFF 5ml column (GE Healthcare). The peak from the gradient elution with 100% of buffer B (25 mM Na-HEPES pH 7.5, 500 mM NaCl, 250 mM Imidazole, 2.5 mM CaCl_2_, 0.1% α-monothyoglycerol) was concentrated on 10 kDa cut-off Amicon Ultra filter units (Millipore) to approx. 3 ml. This sample was applied on a size-exclusion (SEC) column Superdex75 HR16/60 (GE Healthcare) equilibrated with buffer C (25 mM Na-HEPES pH 7.5, 500 mM NaCl, 2.5 mM CaCl_2_, 0.1% α-monothyoglycerol). The central fractions of His-SUMO-calumenin were pooled and treated with SUMO-protease (1:10 molar ratio) overnight at 4°C.

**Fig 2:**
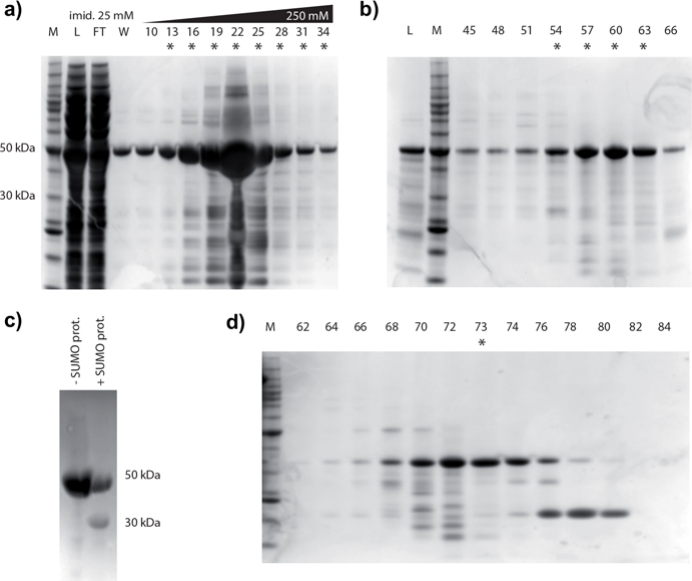
SDS-PAGE overview of calumenin purification. His-tagged calumenin was first isolated from the lysate using a HisTrap affinity column (a). The eluted protein was further purified on a SEC column (b). The resulting tagged protein was submitted to SUMO-protease digestion (c), and the tagged sample (- SUMO-prot.) was treated until the formation of a 30 kDa band in the protease-treated sample (+ SUMO-prot.). Reverse affinity was used to eliminate any uncleaved protein and the tag (not shown). Finally, the now untagged calumenin was purified on a second SEC column (d). M = Benchmark molecular weight markers, L = loaded sample, FT = unbound flow-through fraction, eluted fractions are indicated with cardinal numbers. For the column, these numbers correspond to the retention volume (in ml). Fractions indicated with an asterisk were kept for the next stages of purification.

The cleaved calumenin (Fig 2C, + SUMO-prot.) was separated from the tag, the undigested protein and the protease (Fig 2C,—SUMO-prot.) through reverse IMAC on a HisTrapFF 5ml column. The flow-through fraction, concentrated as above was submitted to size-exclusion chromatography and the peak concentrated in buffer D (25 mM Na-HEPES pH 7.5, 250 mM NaCl, 2.5 mM CaCl_2_) to approx. 8.82 mg/ml. 15 μl aliquots were flash frozen in liquid nitrogen and stored at -80°C.

### Analytical gelfiltration

Analytical gel-filtration profiles of calumenin were obtained in the presence (+ 2.5 mM) or in the absence of CaCl_2_ using a Superdex 200 Increase 10/300 GL column (GE Healthcare) equilibrated with buffer E (25 mM Tris-Cl pH 7.5, 150 mM NaCl, 2.5 mM CaCl_2_) and F (25 mM Tris-Cl pH 7.5, 150 mM NaCl, 2.5 mM EGTA) respectively. The same column was equilibrated with buffer G (25 mM Tris-Cl pH 7.5, 150 mM NaCl, 2.5 mM SrCl_2_) to evaluate the effect of Sr^2+^.

Elution peaks were also submitted to multi-angle light scattering (MALS), dynamic light scattering (DLS) and refractive index (Ri) analysis (DAWN HELEOS II + Optilab T-rEX, Wyatt) and data were processed through the software package ATLAS 5 to estimate the molecular weight and hydrodynamic radius.

### Circular dichroism experiments

The concentrated protein solution, stored at -80°C in 300 μM aliquots, was thawed on the day of the experiments and diluted to 100 μM using a buffer H (25 mM Na-HEPES pH 7.5, 50 mM NaCl, 10 mM EGTA) to strip the Ca^2+^ from the protein. Both Ca^2+^ and the chelating agent were removed from solution by loading 200 μl of the protein solution on a 5 ml HiTrap desalting column (GE Healthcare) equilibrated in buffer I(25 mM Na-HEPES pH 7.5, 150 mM NaCl).

The apo-protein was further diluted to 5 μM with buffer I or J (25 mM Na-HEPES pH 7.5, 150 mM NaCl) to a final NaCl concentration of 25 mM (for low ionic strength experiments) or 150 mM (for physiological ionic strength experiments).

Circular dichroism spectra were collected in the far-UV region (185–260 nm) using a 0.05 mm pathlength quartz cuvette kept at 20°C at different Ca^2+^ concentration points. These were achieved by small additions of concentrated CaCl_2_ to the original solution, using three stock solutions obtained by serial dilution of a CaCl_2_ standard (Fluka 21049). All CD spectra were obtained from quadruplicate measurements, averaged and buffer subtracted.

The CD titration experiments [14] were performed using Module B of the beamline B23 of Diamond Light Source and analysed with the software CDApps [15]. Cooperativity of binding was assessed by fitting the experimental data with a Hill function through the software Origin (OriginLab).

### Surface plasmon resonance

Surface plasmon resonance (SPR) experiments were performed on a Biacore T200 instrument (GE Healthcare). A sensor-chip CM5 was functionalised with calumenin in the presence of 1 mM CaCl_2_ at pH 4.5 according to the manufacturer’s protocol to obtain a response of approximately 2000 a. u. during the binding phase.

The binding experiments were performed by flowing solutions containing different concentration of the cations in 1x HBS-N buffer (GE Healthcare). At the end of each cycle the residual metals were stripped from the chip with a wash of HBS-N buffer supplemented with 5 mM EDTA followed by a re-equilibration in HBS-N.

All binding experiments, performed in quadruplicate, were averaged and analysed with the Biacore analysis software.

### Small-angle X-ray scattering

Preliminary small-angle X-ray scattering (SAXS) data were collected on samples at different concentration of calumenin both in the presence and in the absence of Ca^2+^ at the beamline I22 (Diamond Light Source). Averaging of repeated scans and background subtraction were performed with the DAWN software package.

SAXS data collection coupled to SEC was performed at beamline B21 (Diamond Light Source) using a Shodex PROTEIN KW-803 size-exclusion column loaded with buffer D from the purification. The scattering curves from 40 data points within the chromatography peak were scaled and averaged to obtain the I(q) function and the same region from a blank run (no protein loaded), was used to estimate the background scattering.

Data reduction and preliminary analysis were performed with the software ScÅtter [16].

Since the radial scattering profile of Ca^2+^-free calumenin was compatible with that of an unfolded protein (pure scattering); further analysis was not performed on the apo-protein datasets.

For the Ca^2+^-bound calumenin, estimation of R_g_ was obtained by the AutoRg module of the ATSAS suite [17] through linear regression of the ln(q) vs q^2^ plot of 180 points at low q. The shape of the protein was reconstructed using DAMMIF [18] and 24 of these calculations were averaged with Damaver [19] to provide the final envelope.

### Structure predictions

Secondary structure prediction was obtained through the Xtalpred server [20].

Manual alignment of the EF-hand domains was performed according to Rogstam *et*. *al* [21].

Three dimensional structure prediction was performed by submitting the amino acid sequence of calumenin (68–315) to the servers iTasser [22], Phyre2 [23] and SwissModel [24].

Calculation of R_g_ from the coordinate files of the different predictors was performed using the software Hydropro [25].

The highest ranking models from the predictors above were superposed to the SAXS envelope using the software Supcomb [26].

## Results

### Recombinant calumenin expression and purification

Secondary structure prediction analysis of the calumenin precursor gene (Uniprot ref. O43852) using different algorithms identified six Helix-Loop-Helix (HLH)-motifs downstream of the N-terminal signal peptide. All six motifs contain the hallmarks of the EF-hand Ca^2+^ binding motifs, bearing a canonical loop sequence (DxDxDGxxxxEE) within two alpha-helices. We also identified a seventh loop sequence upstream of the first EF-hand domain. This loop (aa. 46–67) resembles a canonical EF-hand motif including aspartate and glutamate residues, but lacking the fundamental glycine residue in position 6 that confers high flexibility to the EF-hand by allowing major rearrangements upon Ca^2+^ binding. The loop also lacks the flanking helical pattern characteristic of the EF-hand domains and was predicted to be in a disordered conformation. We thus defined it an “orphan” loop (Fig 1) which is likely to retain Ca^2+^-binding capabilities [6], even in the absence of secondary structure motifs as seen in other non EF-hand proteins such as calsequestrin.

For this study we have focused exclusively on the six canonical EF-domains and we have cloned and expressed the C-terminus of calumenin in bacteria (aa. 68–315).

We achieved high expression levels of calumenin in *E*. *coli* using the pOPINS vector [12]. The protein, produced as a fusion with His_6_-SUMO tag, was purified through subsequent steps of IMAC, size exclusion chromatography and tag cleavage. Initial attempts to purify the protein in the absence of Ca^2+^ (supplementing most purification buffers with 1 mM EDTA), resulted in very low purity of final sample fractions, which we attribute to the intrinsic “stickiness” of the protein to the bacterial ones as well as degradation issues. To test this hypothesis, we added 2.5 mM CaCl_2_ to all the buffers and performed the purification as described in the Material and Methods section. This approach resulted in much higher purity compared to initial purifications, allowing us to isolate a few pure fractions (fraction 73 in Fig 2D was quantified to be 90% pure, S2 Fig). The SDS-PAGE densitometry analysis displayed fewer faint bands at low molecular weight for fraction 73, compared to those in fractions 74 and 72. For this reason we chose to use the sample from fraction 73 for the structure/function studies presented in this work.

### SEC-MALS

The preparative grade size exclusion chromatography (SEC) profiles of Ca^2+^-loaded calumenin are very different from those of the Ca^2+^ -free protein. The shift in size exclusion retention volumes suggests a major Ca^2+^ -induced conformational change in calumenin.

To investigate this behaviour in greater detail, we loaded a concentrated purified sample of calumenin(68–315) on an analytical Superdex 200 Increase 10/300 GL column coupled to a setup for measuring MALS/DLS/Ri.

We analysed calumenin with and without Ca^2+^ (2.5 mM CaCl_2_ and 2.5 mM EGTA in the column buffer respectively), and the system was equilibrated thoroughly before each run to allow precise Ri measurements.

**Fig 3:**
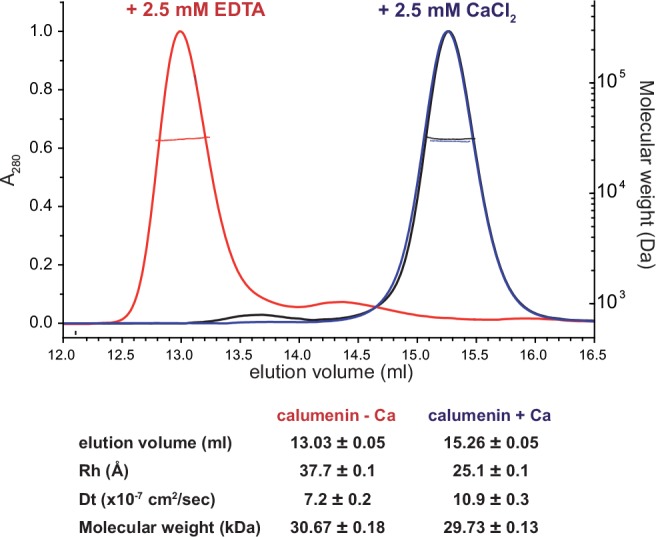
Ca^2+^-induced shift in size exclusion chromatography. Comparison between the SEC-MALS profiles of calumenin in the presence (blue and black) or in the absence (red) of Ca^2+^. The dashed lines crossing the peaks represent the MALS calculated molecular weight (right vertical axis). Calculations for the hydrodynamic radius (R_H_) and molecular weight are provided in table for the two peaks.

The chromatograms, shown in Fig 3, displayed a large peak shift where apo-calumenin(68–315) has a lower retention volume, compared to the Ca^2+^-bound form.

The elution volumes are calculated by fitting the whole peaks to a Gaussian profile over 100 data points (n = 100) and are given in the figure as mean values and standard deviations. A t-test confirms the difference between the two populations is extremely statistically significant (P≤0.0001)

MALS measurements on the two peaks confirm that in both cases the protein is in its monomeric state, which rules out multimerisation/aggregation, in contrast with calsequestrin, which oligomerises in the presence of mM concentrations of Ca^2+^. The values of R_H_, D_t_ and molecular weight (MW) reported in Fig 3 were obtained by averaging the 40 values calculated by the software ASTRA V (Wyatt Technology) for the central regions of each chromatographic peak (n = 40) on the basis of MALS/DLS/Ri measurements.

We also treated a sample with 2.5 mM CaCl_2_, subsequently stripped with 2.5 mM EGTA and injected in a column equilibrated with the buffer containing 2.5 mM CaCl_2_. The profile (in black in Fig 3) almost completely overlaps with the protein not subjected to Ca^2+^ stripping confirming the Ca^2+^-dependent condensation is a reversible phenomenon.

The experiment was also repeated by adding to the protein 2.5 mM Sr^2+^ to verify if this ion, a common mimic of Ca^2+^, could induce similar effects on the elution profile of calumenin (S3A Fig). Surprisingly, the retention volume differed from the previous two cases (apo- and Ca^2+^-bound in Fig 3) and the elution peak was found at an intermediate position. In addition to this, a peak in correspondence to the void volume of the column was observed when Sr^2+^ added to the buffer instead of EGTA or Ca^2+^.

DLS analysis allows the calculation of the hydrodynamic radius (R_H_) of the proteins in the two peaks. The R_H_ value of 25.1 ± 0.1 Å found for the Ca^2+^-bound calumenin(68–315) is compatible with a slightly elongated protein of approx. 30 kDa. For the apo-calumenin, the much higher value of R_H_ (37.7 ± 0.1 Å) suggests that a highly disordered/unfolded structure is present in the absence of Ca^2+^.

### Circular dichroism

To further characterise the Ca^2+^-induced transition of calumenin, we analysed the protein using SR-CD. This would allow us to detail the Ca^2+^ dependence of the transition as well as the nature of the conformational changes leading to a compact Ca^2+^-bound form.

To our great surprise, the spectrum of calumenin(68–315) in the absence of Ca^2+^ is that of a disordered protein, with no detectable degree of alpha-helices. However, the same sample treated with mM concentrations of Ca^2+^ increases the alpha-helical content substantially. The transition could be reverted by stripping Ca^2+^ with EGTA, accounting for a reversible rearrangement of calumenin in response to different concentrations of Ca^2+^ similar to what observed in SEC-MALS experiments (Fig 3).

To further elucidate the mechanism, we performed CD-titration experiments with calumenin which had been purified in the presence of Ca^2+^, treated with EGTA and finally re-buffered through a desalting column (HiPrep desalting, GE Healthcare) to eliminate any traces of Ca^2+^and EGTA.

CD spectra were collected on these samples after additions CaCl_2_ solutions and the data were analysed with the dedicated software CDApps [15]. The spectrum of apo-calumenin presents the typical features of a disordered protein with a negative peak at 200 nm (S4A Fig, curve A). Upon addition of Ca^2+^, the spectrum changes to that of a typical alpha-helical protein and displays a positive peak at 192 nm, a negative doublet at 208 nm and 219 nm and ellipticity = 0 at 200 nm (S4A Fig, curve B).

**Fig 4:**
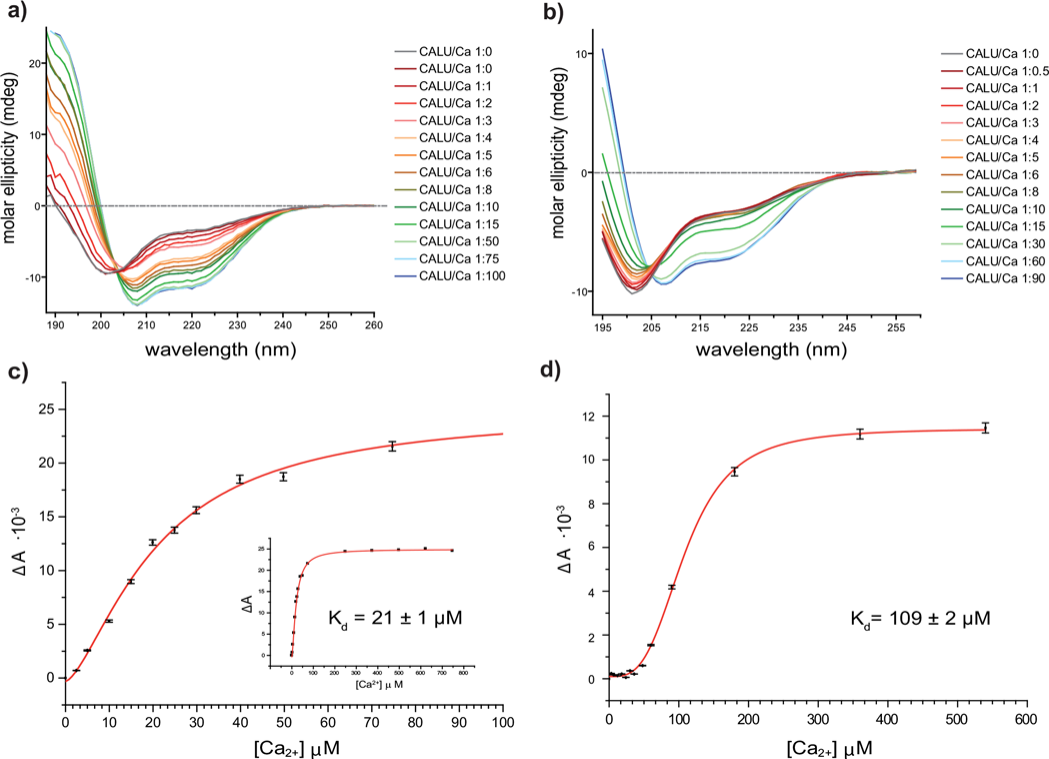
Far-UV CD titration of calumenin. Far-UV CD spectra for the titration of calumenin with increasing concentrations of Ca^2+^ at 25 mM NaCl (a) and at 150 mM NaCl (b). Plot of ΔA_(219 nm)_ vs Ca^2+^ at 25 mM NaCl. Curve fitting was used to calculate the K_d_ value for Ca^2+^ binding to calumenin. The whole experimental range is reported in the inset (c) The same folding plot is provided for the titration at 150 mM using the CD value at 209 nm as a reporter of protein folding.

The formation of secondary structure was monitored as molar ellipticity at 219 nm in the case of the spectra collected at 150 mM NaCl. For the experiments at 25 mM NaCl, the peak at 192 nm was considered. Based on the graphs in Fig 4A and 4B, we report the degree of conformational change versus the Ca^2+^ concentration in plots Fig 4C and 4D. Each data point in these graphs resulted from the average value from four CD spectra and 14 concentrations of Ca^2+^ were used to calculate K_d,Ca_ values through non-linear fitting algorithms (n = 14). Based on the graphs in Fig 4A and 4B, we report the degree of conformational change versus the Ca^2+^ concentration in plots Fig 4C and 4D. A clear transition between an unfolded to alpha-helical structure occurs in the range studied and has been estimated to have a K_d,Ca_ of 21 ± 1 μM (n = 14) at buffer salt concentration of 25 mM NaCl. Increasing Ca^2+^ concentration leads to a marked loss in turns/disorder content (from 58% to 40%) and a corresponding increase of alpha helix content (from 17% to 50%).

Secondary structure estimation from CD spectra was calculated using the CONTIN/LL algorithm [27] in CDApps [15] by deconvolution of the whole spectra, *i*.*e*. the percentage of alpha-helical structure was obtained considering all 71 CD data points at a given Ca^2+^ concentration (S4B Fig). When the same system is studied at physiological salt concentration (150 mM NaCl), the K_d,Ca_ increases to 109 ± 1 μM (n = 14) and the increase of alpha helix content is slightly reduced (~35%) compared to the low salt condition.

Calumenin requires Ca^2+^ to form a secondary structure either at high or low NaCl concentrations, but the final folded states are different in the two cases (Fig 4A and 4B). The shapes of red curves (unfolded state) are approximately the same, but the blue curves (folded state) are intrinsically different, displaying a less pronounced feature at 208 nm in the CD spectra obtained at high salt concentration.

To determine the Ca^2+^ specificity of this conformational change we repeated similar measurements with Sr^2+^ but we only observed a partially folded alpha-helical structure even at 1 mM Ca^2+^ (S3 Fig).

### Surface plasmon resonance

A further confirmation on the Ca^2+^ binding activity of calumenin(68–315) was investigated using SPR. The protein was immobilised on a CM5 chip (GE Healthcare) either in the presence or in the absence of 2.5 mM Ca^2+^ in the immobilisation buffer.

**Fig 5:**
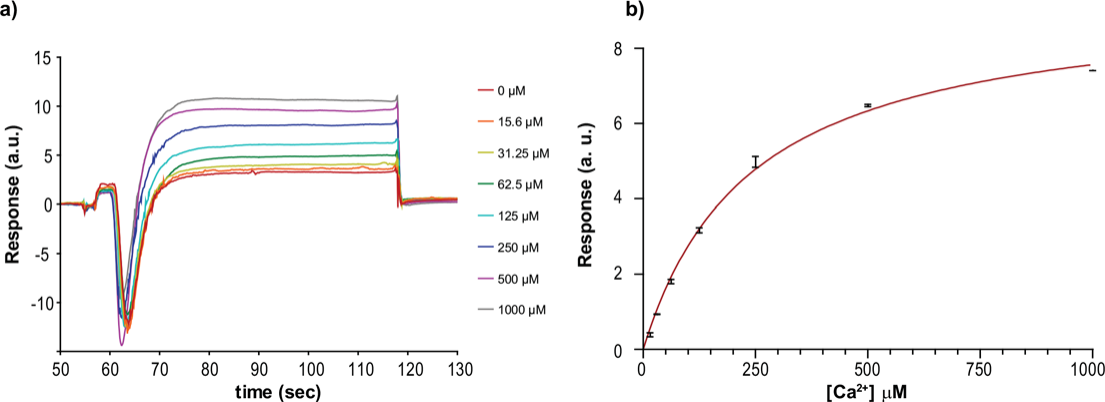
Surface Plasmon resonance of calumenin with different divalent ions. SPR analysis of Ca^2+^-binding to calumenin. Sensorgrams obtained at 8 different ion concentrations (a) in quadruplicate were processed with the Biacore analysis software. The curve (b) provided a K_d,Ca_ value of 201 ± 13 μM (n = 32) for the interaction between Ca^2+^ and immobilised calumenin.

To avoid Ca^2+^ precipitation, we used the 1xHBS-N buffer provided by the manufacturer. Serial dilutions of Ca^2+^, Sr^2+^, Mg^2+^ and Mn^2+^ were prepared and used on the calumenin(68–315)-immobilised chip to quantify the binding constant of the ions. An equilibrium analysis confirms that Ca^2+^ is the only ion in the panel binding the protein in the sub millimolar range. The k_on_ and k_off_ for Ca^2+^ were extremely fast which, combined with the low signal and the anomalous post-injection peak shape, did not allow us to make a full kinetics analysis (Fig 5). This very noisy post-injection signal may be partly attributed to the extensive conformational changes of calumenin upon Ca^2+^ binding.

The binding of other ions with comparable cation radius ratio to Ca^2+^ was also evaluated by SPR, but did not give sufficiently low K_d_ values upon fitting. Values above 1M are indicative of aspecific binding and the effect of these ions, apart from Sr^2+^, was not investigated further (S5 Fig).

### Small-angle X-ray scattering

**Fig 6:**
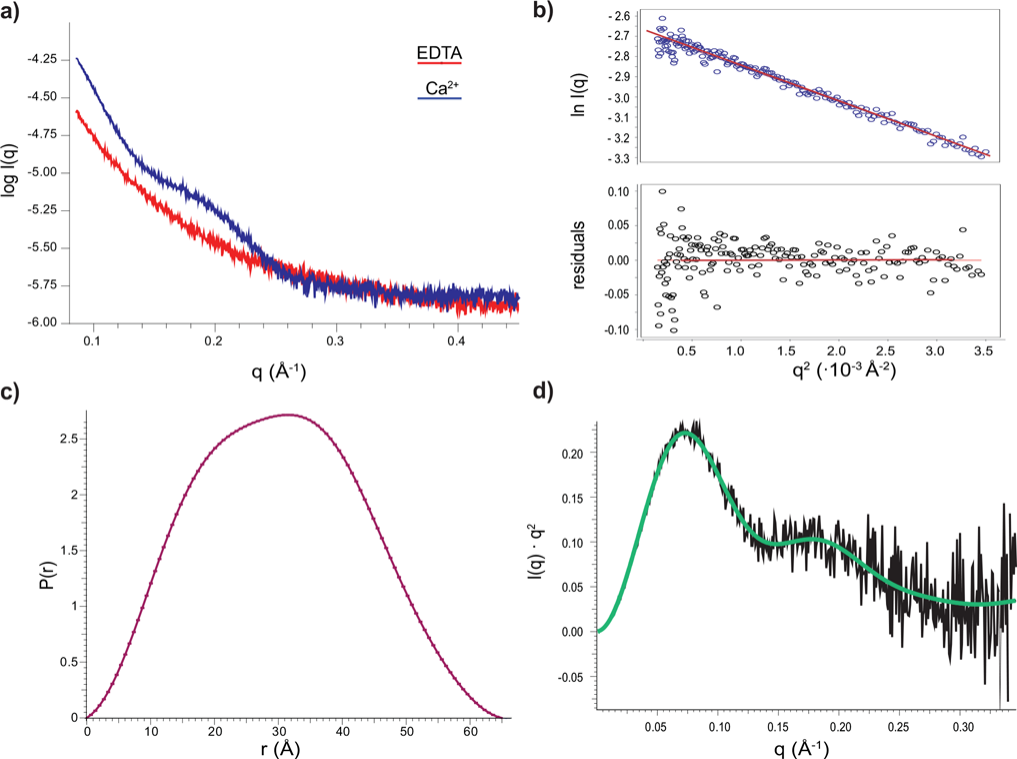
Small-angle X-ray scattering of calumenin. SAXS experimental I(q) curves for apo- (red) and Ca^2+^-bound calumenin (blue) (a). The slope of the Guinier plot, i.e. the ln(q) vs q^2^ function at low q provides the radius of gyration R_g_ of the molecule (b). The P(r) function representing the distribution of the length of interatomic vectors (c) and Kratky plot(d) were used respectively to calculate the shape of the molecular envelope and to estimate the flexibility of calumenin.

To provide a low-resolution structure of calumenin(68–315) in its Ca^2+^-bound state, we collected SAXS data from solutions of the protein at different concentrations at the I22 beamline of Diamond Light Source. The SAXS data confirmed that apo-calumenin is likely to be unfolded, as the scattering profile recorded resembles that typical of a random coil peptide chain (Fig 6A, red curve), and we did not progress with detailed analysis of this sample. Instead we focussed on that of the Ca^2+^-bound calumenin(68–315), which presents features for around q~0.1Å^-1^ which are indicative of the presence of small structured objects in the range of the 10s of Å (Fig 6A, blue curve).

However, a detailed analysis of the diffraction images showed a severe radiation damage effect most likely due to a combined effect of the high flux delivered by the insertion device beamline and the long exposure time over the same portion of sample. To minimise both effects to the sample, we repeated the measurements using the HPLC-SAXS setup available at the B21 beamline of Diamond Light Source. SAXS images were obtained from the eluted peak so that each image was collected from protein not previously exposed to X-rays. This procedure enabled us to obtain high quality and highly redundant data (scatter profile averaged on 40 images). We calculated the radius of gyration of the molecule (R_g_) applying the Guinier law [17] to the low q regime of the averaged ln(q) vs q^2^ curve (Fig 6B). The R_g_ value of 22.8 ± 0.3 Å (n = 40) agrees with the R_H_ value of 25.1 ± 0.1 Å (n = 40) estimated from the R_i_ analysis described above. The calculated R_g_ of 2.28 for the Ca^2+^-bound protein gives an R_g_/R_H_ ratio = 0.908 compatible with an elongated structure (0.775 < R_g_/R_H_ < 1).

An envelope for calumenin was calculated from the P(r) function, the distribution of the lengths of interatomic vector lengths obtained by Fourier transform of the scattering curve (Fig 6C). We obtained a bell-shaped curve with a small shoulder, as often encountered for multidomain proteins. The final envelope was calculated from the average result of 24 simulation obtained with the software DAMMIF [18].Structure prediction and combination with experimental data

To interpret the SAXS envelope described above we searched for possible models by submitting the protein sequence of calumenin(68–315) to online servers dedicated to the prediction of 3D structures of proteins. We tested results obtained using Swiss-Model [24], Phyre2 [23] and iTasser [22]. All of them provided one or more hits covering the entire 6 EF-hand domains of the protein. All the predictors failed to model the N-terminus of the protein given no homologous sequence is found in the PDB. The limited set of solutions proposed by the servers is most likely a consequence of the limited number of structures of hexa-EF hand proteins and the lack of high identity templates in the PDB. The most homologue template used was the protein secretagogin from *D*. *rerio*, a neuronal cytosolic protein involved in stress hormone release which shares only 21% homology with calumenin [28,29].

All the models proposed arranged twelve helices in non-globular structures, those based on secretagogin (PDB 2q4u) being the most elongated. To rank the protein predictions, we estimated the R_g_ from the corresponding coordinate files using the software Hydropro [25].

The highest ranking predictions from Phyre2, iTasser and SwissModel, displayed R_g_ values of 19.7 Å, 21.0 Å and 22.0 Å respectively, values comparable to the experimental value of 22.3 Å obtained from our HPLC-SAXS experiments. We fitted the protein envelopes calculated for our SAXS experiments on Ca^2+^-bound calumenin(68–315) with the models generated using the above servers using the software Supcomb [26].

The structure predicted by Phyre2 was too compact to fit the experimental envelope, failing to occupy the most peripheral zones, while bulging out of the central zone in portions of the space for which no electron density was observed. Manually fitting the model to the experimental data was not successful either: while one portion of the model could be forced to overlap the envelope, the orientation of the remaining part was unsatisfactory (S6 Fig)

**Fig 7:**
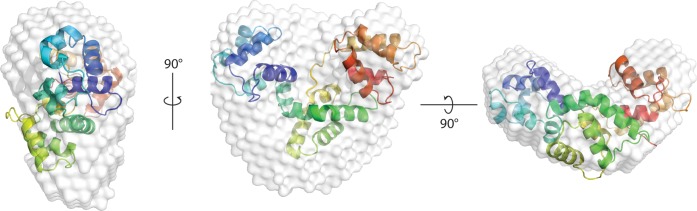
Calumenin 3D structure prediction and fitting with experimental data. Overlap of the calumenin model obtained from iTasser (coloured ribbons) with the envelope calculated from the SAXS data (white volume). Three orthogonal projections are shown confirming a good concordance between the trilobal model of calumenin and the experimental data.

We obtained much more convincing fits with the structure predicted by iTasser and SwissModel which overlapped almost perfectly to the SAXS model (Fig 7 and S1 Video). Each of these models consists of three lobes formed by two adjacent EF-hand domains, in a slightly elongated fashion. Small portions between these lobes are still present in the SAXS envelope, but not in the model, suggesting some degree of protein flexibility. This is also confirmed by the Kratky plot (Fig 6D), which analyses the degree of compactness and foldedness of proteins [4]. In the case of a perfectly globular and compact protein, the plot is a bell-shaped curve asymptotically going to 0 for high values of q, whereas totally unfolded proteins plateau at large q values. In the case of calumenin, the plot presents more features than for a globular protein and at high q shows an intermediate behaviour between the two extreme states, suggesting a partial degree of flexibility for the protein.

## Discussion

The EF-hand domain is a fundamental functional unit designed by nature to transduce Ca^2+^ signal via structural rearrangements of helices around a Ca^2+^-chelating loop. A prototypical member of the EF-hand containing protein superfamily is calmodulin (CaM), a protein that undergoes major structural changes in response to variations in cytosolic Ca^2+^ concentration. This Ca^2+^-sensing protein selectively activates numerous biological processes, including Ca^2+^ homeostasis through interactions with the Plasma Membrane Ca^2+^ATPase (PMCA). Binding of Ca^2+^-loaded calmodulin to PMCA release autoinhibition and activates the pump. Calumenin has also been found to regulate a primary Ca^2+^ transporter, albeit with a different functional outcome. Binding of Ca^2+^-loaded calumenin to SERCA2a reduces the apparent Ca^2+^ activity of the transporter. Findings that calumenin also binds the ryanodine receptor which releases Ca^2+^ from the SR to induce muscle contraction, puts calumenin as a key player in the regulation of the SR and cellular Ca^2+^ homeostasis.

Very little is known about the organisation of the domains of the hexa-EF hand proteins, and no high-resolution structure is available so far for any of the CREC family members, to which calumenin belongs. Despite the high yields of calumenin obtained from heterologous expression in *E*. *coli*, crystallisation has been hampered by a number of factors such as (a) its propensity to co-elute with other bacterial proteins and to degrade (b) its low pI and (c) its intrinsic flexibility. The structures of cytosolic hexa EF-hand proteins have been determined [28,30], but their sequence identity with the members of the CREC family is very low. Adding to that the significant difference in Ca^2+^-affinities compared to the EF-hand proteins located in the secretory pathway, suggests that cytosolic EF-hand proteins may have a different structural arrangement.

Here we have focused on characterising calumenin, which has previously been reported to bind the L4 loop of SERCA2a through hydrophobic interactions with the domains EF3 and EF4 [31]. This interaction was only observed at high Ca^2+^ concentrations, so the authors concluded that this SERCA2a-calumenin interaction occurred when the pump was in its E1 conformation. The structural alignment of the close homologue SERCA1a in all the states of the catalytic cycle does not explain the preference for the E1 state over the E2 since the L4 loop, although highly flexible, always appears in similar conformations. This large solvent-exposed loop, one of the most flexible regions of SERCA1a, as reflected by the high B-factor values, cannot completely justify the binding, unless calumenin can provide such type of conformational selection. As all EF-hands domains, calumenin was thought to change the arrangement of alpha-helices in response to local Ca^2+^ concentration changes and, as such the Ca^2+^-bound form of calumenin could stabilise the L4 of SERCA2a in one of its E1 conformations.

Overall our results suggest that Ca^2+^-binding to calumenin provides the structural determinant essential to interact with Serca L4 loop in possibly any conformation (E1 or E2 sub-states).

Differently from other EF-hand rich proteins, which only rearrange their helical content upon binding Ca^2+^, calumenin exists in a random coiled apo-form. We have shown through CD titration that this converts into a predominantly alpha-helical structure by increasing the concentration of Ca^2+^ to the high μM range (K_d,Ca_ = 21 ± 1 μM). Further SPR and SR-CD experiments at physiological ionic strength account for slightly higher values (201 ± 13 and 109 ± 1 μM respectively). These values are lower than those previously found with different techniques, but still consistent with the knowledge that CREC proteins have sub-mM Ca^2+^ affinities [6]. This relatively low affinity for Ca^2+^ should not surprise, since Ca^2+^ concentrations within the ER can be in the mM range [32]. Calumenin can therefore serve as a Ca^2+^ sensor, capable of regulating molecular partners when the ER is loaded with Ca^2+^, *i*.*e*. when the amount pumped into the ER by SERCA2a reaches the mM range.

Differently from other Ca^2+^-sensing proteins such as calsequestrin which can bind other cations such as Mn^2+^ and Mg^2+^ [33], EF-hand proteins tend to be more specific towards Ca^2+^ (or the very close mimic Sr^2+^). In the case of calmodulin, for instance, Sr^2+^ binds to Ca^2+^ sites, but the resulting structure is locked in a transition intermediate between the apo- and the Ca^2+^-bound state [34].

The specificity of calumenin for Ca^2+^ appears even higher, since mM concentrations of Sr^2+^ only induced a partial alpha-helical conformation to the protein (S3B Fig). This partially folded state was also observed to increase the amount of higher order aggregates which, in size-exclusion chromatography, elute at the void volume of the column (S3A Fig). The different susceptibility of proteins to ions with different charge/radius ratios might reflect the capacity of each cation to assist the folding process and hold together the structure at various stages (*e*.*g*. anchoring the Asp/Glu rich loops, masking acidic stretches during the folding, altering the hydrogen bonding network of the aqueous environment). Similarly to what is observed for calmodulin [34], Sr^2+^ is only partially capable of inducing folding in calumenin. However, since this protein lacks pre-formed alpha-helical structures, Sr^2+^ is probably insufficient to induce the formation of an alpha-helical backbone (S3A Fig) and the protein fails to achieve a compact tertiary structure (S3B Fig)

The existence of two flavours of EF-hand proteins, pre-formed and binding induced, possibly provides an extra regulatory level. Extensive molecular-dynamic studies of the potential underlying mechanisms would be very interesting and we believe our findings on calumenin could provide an excellent case study to define the parameters involved in ion-assisted folding of proteins further. Our CD titration studies on calumenin show that the alpha helical content of the Ca^2+^-bound protein is lower under physiological conditions (150 mM NaCl) than in a low ionic strength buffer (25 mM NaCl). This not only accounts for the aforementioned differences in K_d_ values, but also for the different degree of cooperativity observed. The Hill coefficient indicates high positive cooperativity (n ~ 3.2) for calumenin titration conducted at physiological NaCl concentrations, similarly to what Iida and Potter observed for calmodulin [35].

When the NaCl concentration is lowered to 25 mM, the Hill coefficient decreases (n ~ 1.2) as does the K_d,Ca_, whose value also changes from 109 μM to 21 μM.

In accordance with the job plot obtained for our titrations, where the stoichiometric ratios of ~ 0.65 (i.e 2:1 Ca^2+^ to calumenin) and ~ 0.8 (i.e 4:1 Ca^2+^ to calumenin) in 25 mM and 150 mM NaCl buffer respectively show the availability of different sets of Ca^2+^ sites (S7 Fig).

This accounts for the presence of a calumenin_Ca,I_ intermediate state with high affinity for Ca^2+^, in which only one of the two sites in each pair of EF-hand domains is occupied by the ion. Upon filling the first site, affinity of the second one is increased and a fully-loaded calumenin_Ca,II_ state can be achieved in which both sites of an EF-hand pair are coordinating Ca^2+^ [36]. Ca^2+^ binding to calumenin monitored by CD spectroscopy further showed that under physiological conditions, there is a possibility of further Ca^2+^ ions binding to calumenin.

Further CD and SPR experiments have shown how this process is Ca^2+^ specific and no other divalent ions of comparable ionic radius were able to bind to calumenin(68–315) and to induce reversible folding.

The conformational switch determines not only a major rearrangement of the secondary structure, but also the formation of a compact three-dimensional shape. This has been shown both by our analytical SEC analysis and by SAXS measurements. The apo-form of calumenin(68–315) gives extremely high R_H_ values for a protein of this size and a pure solution X-ray scattering profile, together with the disordered CD results confirm that the protein is intrinsically unstructured in its Ca^2+^-free conformation. Upon Ca^2+^-binding, calumenin(68–315) converts into a more compact protein in which secondary structure elements are arranged in an elongated shape that is compatible with 3D structure predictions.

This is very different from calmodulin, another EF-hand Ca^2+^-binding protein, which in the absence of Ca^2+^ retains the secondary structure elements which are rearranged in space upon Ca^2+^ binding [37,38,39]. Calsequestrin, another Ca^2+^-binding protein also has a preformed core, held together by hydrophobic interaction, and rearranges in space at mM concentration of the ion to form multimeric structures [2,40,41,42].

In the model best matching the experimental SAXS envelope, adjacent pairs of EF-hand domains are arranged in three separate lobes in a modular fashion. Such a model is in accordance with the observation that different proteins bind to different regions of calumenin. In particular the ryanodine receptor recognizes directly the EF-2 domain[10], whereas SERCA2a has been described to bind EF-3 and EF-4 domains of calumenin. We can conclude that the model proposed here for calumenin(68–315) will allow it to interact with the two main Ca^2+^ homeostasis regulators of the ER as proposed. The presence of a third module (EF-5 and EF-6), which has not yet been fully characterised, opens the possibility that besides its Ca^2+^ sensor function, calumenin might act as a docking platform for more complex Ca^2+^ signalling within the ER.

## Supporting Information

S1 Fig Features of the CREC family members. Primary structure alignment of the members of the CREC family with highlight on the Ca^2+^-binding domains of calumenin. The purple box represents the “orphan” EF-hand, while the green boxes indicate the EF1-6 (a). Logo diagram of the EF-hand loop consensus for the CREC family members (b).
(EPS)

S2 Fig Densitometric analysis of purification fractions. Quantitation of the purity of each band from fractions 72, 73 and 74 reported in Fig 2. Numbers above each peak represent the integral of that portion of the densitometric profile (in %).
(EPS)

S3 Fig Effect of Sr^2+^ on calumenin folding. SEC-MALS profiles from Fig 3 were compared with the chromatogram obtained in the presence of 2.5 mM SrCl_2_ (a). SR-CD spectra of the apo-, the Ca^2+^-bound and Sr^2+^-bound forms of calumenin (b).
(EPS)

S4 Fig Ca^2+^ dependency of secondary structure in calumenin. CD spectra of calumenin in 25 mM NaCl (a) obtained in the absence (A) or in the presence of increasing concentrations of Ca^2+^ up to 1:150 molar ratio (B). Each point in (b) was calculated from the deconvolution over all 71 data points of a single CD curve (a) and represents the α-helix or unordered structure contents at a specific calumenin:Ca^2+^ molar ratio.
(EPS)

S5 Fig SPR evaluation of calumenin ion affinity. The figure reports the affinity fit for Ca^2+^, Sr^2+^, Mn^2+^, Mg^2+^ and Cd^2+^ calculated as in Fig 5B. Estimated K_d_ values in the M range, associated with a different ion-dependency profile to that of Ca^2+^, indicate the binding of the other ions is non-specific.
(EPS)

S6 Fig Selection process of SAXS envelope and model superpositions. Visual description of the process adopted to select structure simulation targets to overlap to the SAXS envelope.
(EPS)

S7 Fig Job plots for calumenin-Ca^2+^ titrations. Job plots for the titration of calumenin in the presence of 25 mM NaCl (a) and 150 mM NaCl (b).
(EPS)

S1 Video Overlap of *in silico* model with the envelope from SAXS data. Rotation in 3D of the SAXS envelope superposed to the best fitting *in silico* model obtained.
(ZIP)
